# A streamlined clinical metagenomic sequencing protocol for rapid pathogen identification

**DOI:** 10.1038/s41598-021-83812-x

**Published:** 2021-02-23

**Authors:** Xiaofang Jia, Lvyin Hu, Min Wu, Yun Ling, Wei Wang, Hongzhou Lu, Zhenghong Yuan, Zhigang Yi, Xiaonan Zhang

**Affiliations:** 1 Shanghai Public Health Clinical Center, Fudan University, Shanghai, China; 2 Key Laboratory of Medical Molecular Virology (MOE/NHC/CAMS), School of Basic Medicine, Shanghai Medical College, Fudan University, Shanghai, China

**Keywords:** Biochemistry, Biological techniques, Biotechnology, Diseases

## Abstract

Metagenomic next-generation sequencing (mNGS) holds promise as a diagnostic tool for unbiased pathogen identification and precision medicine. However, its medical utility depends largely on assay simplicity and reproducibility. In the current study, we aimed to develop a streamlined Illumina and Oxford Nanopore-based DNA/RNA library preparation protocol and rapid data analysis pipeline. The Illumina sequencing-based mNGS method was first developed and evaluated using a set of samples with known aetiology. Its sensitivity for RNA viruses (influenza A, H1N1) was < 6.4 × 10^2^ EID50/mL, and a good correlation between viral loads and mapped reads was observed. Then, the rapid turnaround time of Nanopore sequencing was tested by sequencing influenza A virus and adenoviruses. Furthermore, 11 respiratory swabs or sputum samples pre-tested for a panel of pathogens were analysed, and the pathogens identified by Illumina sequencing showed 81.8% concordance with qPCR results. Additional sequencing of cerebrospinal fluid (CSF) samples from HIV-1-positive patients with meningitis/encephalitis detected HIV-1 RNA and *Toxoplasma gondii* sequences. In conclusion, we have developed a simplified protocol that realizes efficient metagenomic sequencing of a variety of clinical samples and pathogen identification in a clinically meaningful time frame.

## Introduction

Historically, laboratory diagnosis of infectious diseases has relied largely on microscopic examination and culture in appropriate media or cell lines. The advent of molecular biological techniques and sensitive RNA/DNA detection-by-amplification methods has dramatically changed clinical practice for infectious diseases^1^. However, these tests require prior knowledge of the infectious agent, and not all molecular tests are readily available for all suspected pathogens in clinical practice. By contrast, metagenomic next-generation sequencing (mNGS) is a bias-free method that retains the key advantages of molecular tests and requires no information on the aetiology of the disease. This method allows the detection of a wide range of microbes (viruses, bacteria, fungi and parasites) present in a sample in a single assay^2–6^. In addition to clinical diagnosis, mNGS has also shown potential in the discovery of novel pathogens, and a case in point was the recent outbreak of infectious pneumonia caused by SARS-CoV-2^7,8^. Thus, mNGS has widespread microbiological applications, including in infectious disease diagnosis in clinical laboratories^9^, pathogen identification for acute and chronic illnesses of unknown origin^10^, and outbreak surveillance on a global scale^7,8,11^.

Despite the significant advantages of the mNGS approach, there are also several technical and regulatory obstacles to this method being widely applied in clinical practice. The most obvious limitation is that the whole process usually takes several days and involves a long chain of wet and dry laboratory activities whose reliability needs to be rigorously validated. In particular, the wet lab procedure usually involves the extraction of minute amounts of nucleic acids, which are subsequently transformed into sequencing-ready libraries with high molecular efficiency. Most of the reported mNGS methods have relied heavily on large amounts of basic research resources and prohibitive expenditure on consumables. This is particularly problematic in resource-poor areas. In addition, although genome sequencing technologies continue to develop with remarkable pace^12–18^, analytical approaches for reconstructing and classifying metagenomes from mixed samples remain limited in their performance and usability^19^. Finally, pre-validated reference databases and sequence analysis pipelines that factor in the common pitfalls of pathogen identification are needed for reliable reporting.

In this study, we attempted to address some of the issues by developing a broadly applicable time- and cost-effective mNGS method. Total nucleic acids from virus stocks and clinical samples, including throat swabs, sputum and cerebrospinal fluid (CSF), were extracted and used to construct separate DNA and RNA libraries, which were further analysed on Illumina or Nanopore sequencing platforms. Our mNGS techniques showed good sensitivity and specificity with reference to conventional clinical tests and helped identify additional respiratory viruses, HIV and *Toxoplasma gondii* from clinical samples.

## Results

### Establishment of an Illumina-based mNGS method

**Figure 1:**
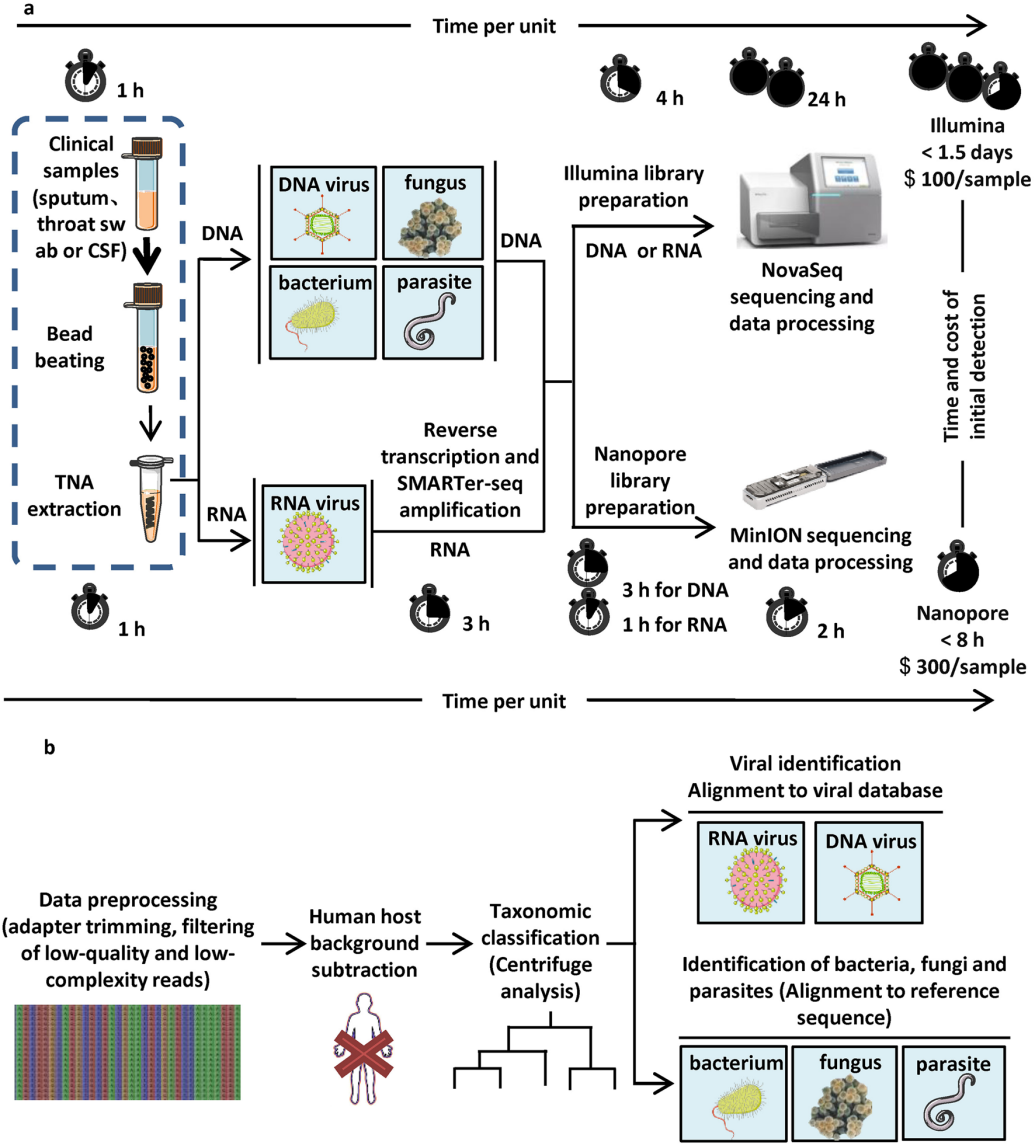
Schematic of the mNGS assay workflow. (**a**) Time-line of the mNGS workflow. Total nucleic acids (TNAs) were extracted by bead beating and guanidinium isothiocyanate-based lysis. TNA was then collected and split into two aliquots for subsequent DNA and RNA library preparation, which were further analysed by Illumina- or Nanopore-based sequencing. The time consumption of each step, the total time spent and the cost estimate of the workflow are indicated. (**b**) Sequence analysis workflow. Sequences generated by Illumina and Nanopore sequencing were processed for alignment and classification. Reads were preprocessed by trimming adapters and removing low-quality/low-complexity sequences, followed by Centrifuge software analysis to taxonomically classify microbial reads into families, genera, or species and alignment to the specific sequence of candidate pathogens.

First, a sensitive and streamlined metagenomic next-generation sequencing (mNGS) protocol was developed and evaluated using a series of virus-positive samples. The general assay workflow is depicted in Fig. 1. Efficient sample lysis was performed using chaotropic salt-based buffer in combination with bead beating, followed by magnetic bead-based semiautomatic nucleic acid extraction. This process required approximately one hour. Another 4 or 7 h were needed for the generation of Illumina sequencing libraries starting from DNA or RNA, respectively. Less than one working day (8 h) is required for an experienced technician to process approximately 20 samples into sequencing-ready libraries. We tested this assay using representative DNA and RNA viruses (HBV-positive serum, human adenovirus type 7 (AdV7), human adenovirus type (AdV3) and influenza A/Puerto Rico/8/1934 H1N1 (PR8)). The resulting sequencing reads (9.86–70.68 million filtered reads) enabled the recovery of full-length viral genomes with average coverage depths ranging from 3262.33 to 12,745.27 (Table 1).

**Table 1:** Illumina and Nanopore sequencing results for positive control samples.

No	Sample	Virus type	Virus titre	Sequencing method	Organism identified from the metagenomic pipeline	No. of filtered reads (× 10^6^)	Total mapped reads	Mapped reads/million (RPM)	Coverage (%)	Ave Coverage depth	Max Coverage depth
1	HBV-positive serum	DNA virus	1.0 × 10^6^ (copies/mL)	Illumina	Hepatitis B virus	9.86	318,866	32,339.35	100.00	12,745.27	73,727
2	Influenza A virus Puerto Rico/8/1934 (H1N1)	RNA virus	3.2 × 10^7^ (EID50/mL)	Illumina	Influenza A virus (H1N1)	70.68	422,426	5976.60	100.00	3262.33	11,954
				Nanopore	Influenza A virus (H1N1)	0.37	13,462	36,384	99.46	407	1598
3	AdV7 virus stock	DNA virus	2 × 10^7^ (TCID50/ml)	Illumina	Human adenovirus type 7	25.41	1,694,032	66,667.92	100.00	6664.40	46,698
				Nanopore	Human adenovirus type 7	0.000022	11	500,000.00	67.50	1.29	4
4	AdV3 virus stock	DNA virus	6.4 × 10^5^ (TCID50/ml)	Illumina	Human adenovirus type 3	23.87	1,266,937	53,076.53	100.00	5079.10	26,655
				Nanopore	Human adenovirus type 3	0.000022	3	136,363.00	23.10	0.23	1

**Figure 2:**
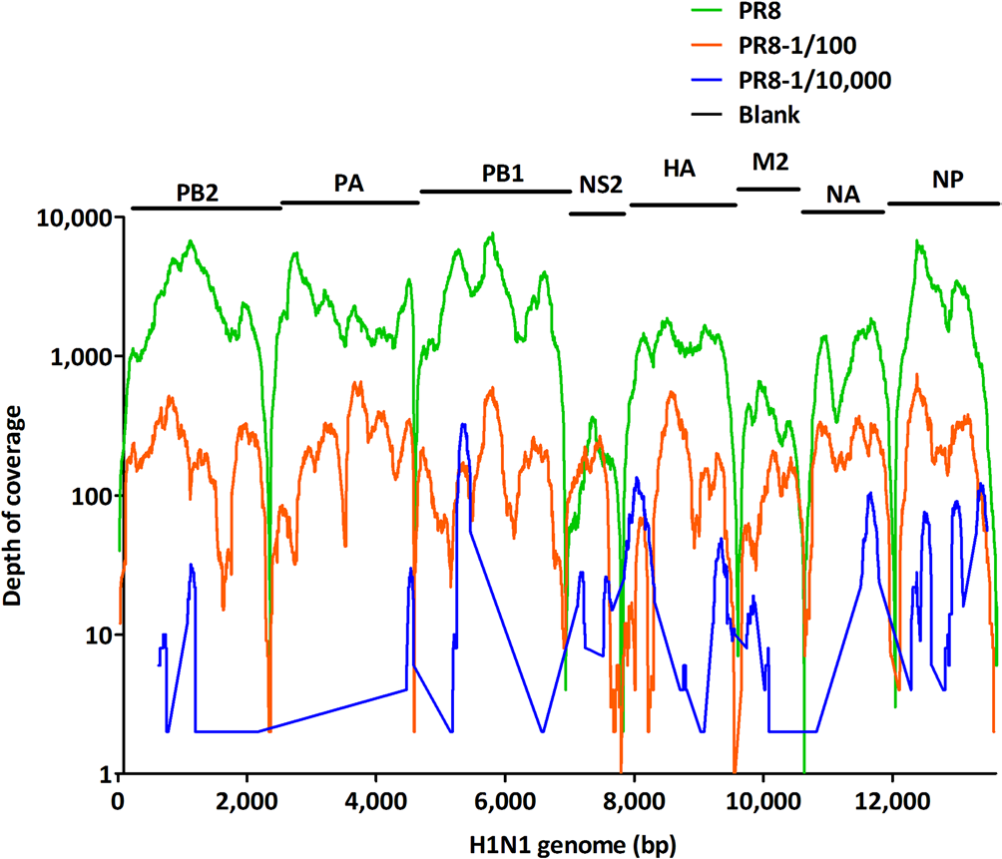
Sensitivity of the mNGS workflow. Genomic coverage from serially (undiluted, 1/100 and 1/10,000) diluted PR8 supernatant and blank control. PR8: Influenza A/Puerto Rico/8/1934 H1N1; PB2, PA, PB1, NS2, HA, M2, NA and NP are the eight segments of the H1N1 genome.

To further assess the sensitivity of virus identification using our method, especially for RNA viruses, a dilution series of PR8 supernatants was tested. While a 1530.02 × (100% coverage) average depth was obtained for the original virus stock, and depths of 174.66 × (90.30% coverage) and 11.98 × (25.60% coverage) were achieved when the virus stock was diluted 1/100 and 1/10,000, respectively (Table 2 and Fig. 2). A good correlation between sample viral loads and the number of total mapped reads was observed (p = 0.02, r = 0.99, linear regression), while reads generated from the negative control showed no mapping. Although the genome coverage of the virus with the highest dilution factor (1/10,000, 6.4 × 10^2^ EID50) decreased to 25.60% with a total of 1605 reads mapped to the PR8 genome, it was still more than sufficient for reliable identification. These results suggested that the limit of detection for PR8 was well below 6.4 × 10^2^ EID50.

**Table 2:** Illumina sequencing results for serially diluted PR8 influenza virus.

Sample no	Sample type	Dilution factor	Virus input (EID50/mL)	No. of filtered reads (× 10^6^)	Total mapped reads	Mapped reads/million	Coverage (%)	Ave coverage depth	Max coverage depth
1	PR8	–	6.4 × 10^6^	20.72	205,036	9895.56	100.00	1530.02	7684
2	PR8	1/100	6.4 × 10^4^	20.45	20,570	1005.87	90.30	174.66	742
3	PR8	1/10,000	6.4 × 10^2^	21.41	1605	74.96	25.60	11.98	325
4	Blank control	–	–	25.59	0	0	0	0	0

### Nanopore sequencing of RNA and DNA viruses

**Figure 3:**
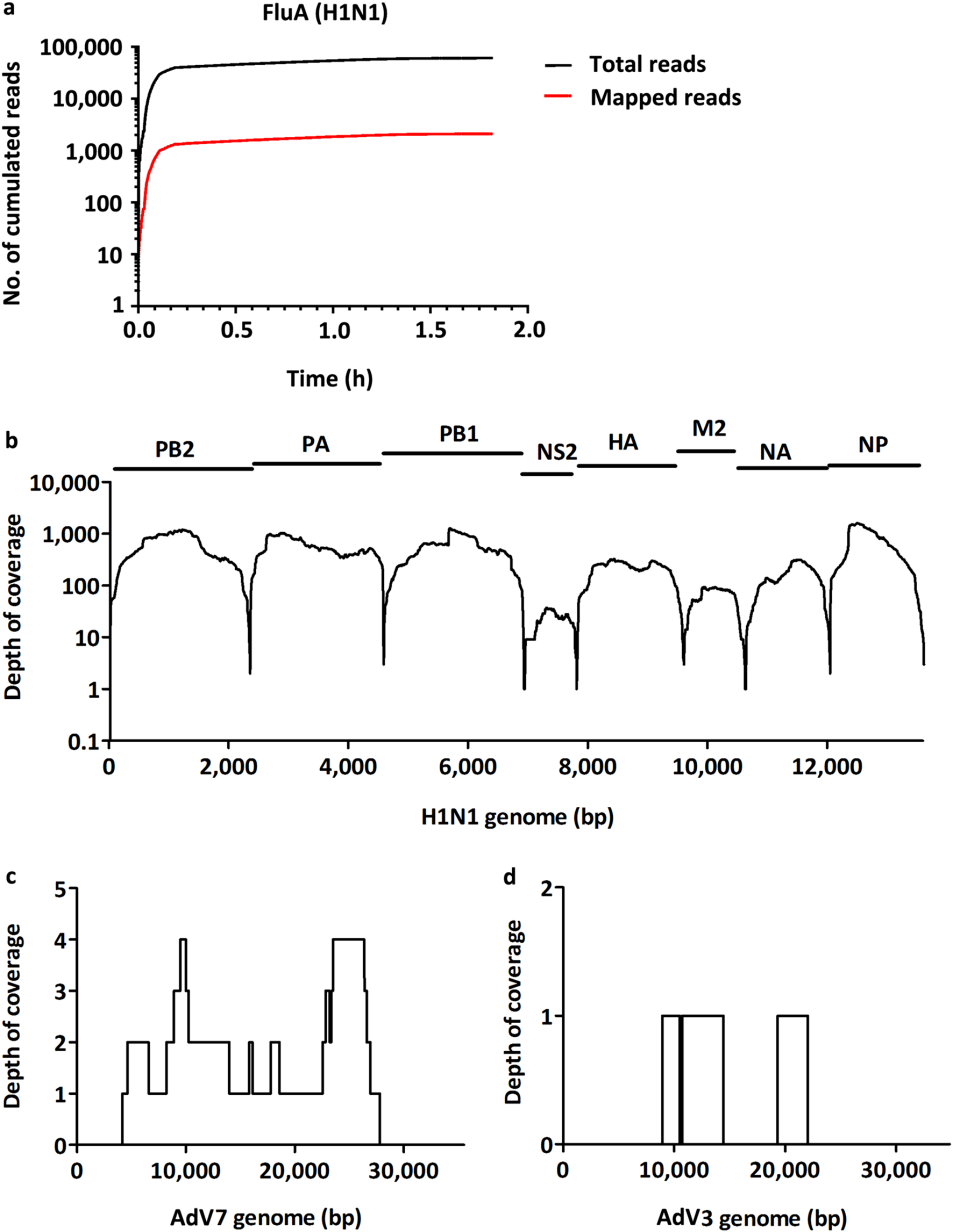
Performance of Nanopore sequencing on selected RNA and DNA viruses. (**a**) Cumulative read plot of H1N1. Genomic coverage plot of H1N1 (**b**), AdV7 (**c**) and AdV3 (**d**).

Single-molecule sequencing technology from Oxford Nanopore has the advantage of real-time data acquisition, which could significantly reduce the overall turn-around time. We first evaluated its performance on influenza A virus using the PR8 stock as a positive control. As shown in the cumulative read plot (Fig. 3a, Table 1), within the first minute, viral reads were sequenced and continued to accumulate. In the first 2 h, 2123 of the total 61,432 reads (3.46% mapping rate) were mapped to one of its eight segments. At the end of the run, 13,462 reads were mapped within 0.37 million reads. Near-full coverage (99.46%) was obtained with an average depth of 407 (Table 1). The genomic coverage plot of PR8 is shown in Fig. 3b. After sequencing the PR8 virus, we washed the sequencing chip and reloaded it with barcoded libraries generated with AdV3 and AdV7 DNA. Although the data generated were low due to inactivation of most of the pores, we still found 11 of 22 reads in AdV7 and 3 of 22 reads in AdV3. With such scarce read data, Nanopore sequencing allowed successful assembly of 67.50% and 23.10% of the genome sequences of AdV7 and AdV3 stocks, respectively (Fig. 3c–d, Table 1). These results reflected the real-time sequencing capability of Nanopore technology.

### Validation with clinical samples

The established mNGS protocols were further tested with clinical samples. Eleven throat swab or sputum samples that had been tested for 41 known respiratory pathogens using TaqMan array card real-time PCR were sequenced (Table 3). DNA and RNA libraries were constructed independently for each clinical sample. RNA or DNA sequencing results with additional matching reads for each sample are shown in Table 3. Among 10 samples that tested positive by the TaqMan array, 8 were positively detected by our mNGS workflow, which included two FluA H1N1, one FluA H3N2, two rhinoviruses, one coronavirus OC43 and two adenoviruses. Sequencing reads from two samples, which were positive for *Haemophilus influenzae* (sample #9) and FluA H1N1 (sample #10), did not meet the statistical criteria for pathogen calling. Sequencing results from one sample (sample #11) that tested negative using the array card did not show significant reads from these pathogens. Thus, our current mNGS sequencing method showed 81.8% (9 in 11) concordance with qPCR-based results. Due to the usually low level of pathogen nucleic acids, these samples yielded 1.00 to 85.29 mapped reads per million (RPM) with a genome coverage of 2.03%-98.75%. For sample #1 with a Ct value of 19.02, near full-length H1N1 viral genomes (98.75%) and an average depth of 13.84 were obtained (Table 3).

**Table 3:** Illumina sequencing results for respiratory and central nervous system samples.

Sample ID	Sample type	Organism identified from clinical testing	qPCR^d^	Microbes identified	No. of filtered reads (× 10^6)^	Lib type	Total mapped reads	Mapped reads/million (RPM)	Coverage (%)	Ave coverage depth	Max coverage depth
1	Sputum	FluA H1N1^a^	19.02	FluA (H1N1)	16.72	RNA	1426	85.29	98.75	13.84	75
2	Throat swab	FluA H3N2^a^	26.74	FluA (H3N2)	9.60	RNA	14	1.46	7.15	0.40	5
3	Throat swab	FluA H1N1^a^	27.16	FluA (H1N1)	18.15	RNA	76	4.19	8.67	1.19	20
4	Throat swab	Rhinovirus^a^	25.99	Human rhinovirus	14.03	RNA	14	1.00	2.03	0.27	4
5	Throat swab	Rhinovirus^a^	32.32	Human rhinovirus	14.20	RNA	539	37.96	62.59	10.57	123
6	Throat swab	Coronavirus OC43^a^	29.31	Human coronavirus	15.90	RNA	582	36.60	8.101	2.53	153
7	Throat swab	Adenovirus^a^	33.13	Human adenovirus B1	21.50	DNA	150	6.98	25.15	0.50	8
8	Throat swab	Adenovirus^a^	24.22	Human adenovirus B1	20.24	DNA	346	17.09	53.66	1.35	21
9	Throat swab	*Haemophilus influenzae*^a^	29.60	N.D							
10	Throat swab	FluA H1N1^a^	26.41	N.D							
11	Throat swab	None^a^	N.A	N.D					–	–	–
12	CSF	HIV-1	7.57 × 10^5^	HIV-1	11.53	RNA	22,964	1425.45	96.73	350.00	1237
13	CSF	*Toxoplasma gondii*^c^	–	*Toxoplasma gondii*	16.11	DNA	290,365	18,024	0.90	0.22	12
		HIV-1	3.14 × 10^4^	HIV-1	13.76	RNA	57	4.14	22.22	0.77	9

^a^Samples tested for respiratory pathogens detected by a customized respiratory TaqMan array card real-time PCR method.

^b^Independent DNA and RNA libraries were prepared for each sample. Data of the indicated library type are listed in this table.

^c^Antibody against *Toxoplasma gondii* tested positive.

^d^qPCR Ct value or virus titres (copies/cell).

We then performed sequencing on 20 CSF samples from patients with meningitis/encephalitis caused by various factors (AIDS-related disease, suspected CNS infection). HIV-1 sequences were identified in two of these patients with mapping rates of 4.14 and 1425.45 RPM (Table 3), and their serum HIV-1 RNA levels were 3.14 E + 04 and 7.57 E + 05 copies/mL, respectively. This confirmed previous reports of cerebral HIV-1 infection in some AIDS patients^20^. Furthermore, *Toxoplasma gondii* sequences (RPMsample = 18,024) were identified in one of these two samples (sample #12, Table 3) with a mapping ratio of 61.7 (RPM-r) compared to the blank control (RPM_NTC_ = 292, data not shown). Indeed, an antibody test for *Toxoplasma gondii* was positive for this patient. This indicated the feasibility of identifying potential parasite infections in CSF samples using our protocol.

## Discussion

The utilization of deep sequencing methodologies in the clinical diagnosis of infectious agents has profoundly improved the speed and precision of infectious disease management in the past decade. In 2014, by shot-gun metagenomic sequencing, Wilson et al.^14^ reported the identification of Leptospira as the aetiology of an unusual case of severe meningoencephalitis, which was one of the earliest examples of the application of this new approach. mNGS has also become a powerful tool for unbiased pathogen detection and monitoring of viral transmission and evolution during outbreaks, which has been best exemplified in the current COVID-19 pandemic^7,8,21,22^. These achievements have highlighted the unique value of deep sequencing for clinical practice and public health intervention.

To fully unleash the diagnostic power of mNGS, tremendous efforts have been made in various key steps of clinical metagenomics, i.e., nucleic acid extraction, library preparation, host sequence depletion, pathogen sequence enrichment, etc.^2,23–30^. However, most of the reported methods have relied heavily on large amounts of basic research resources, entailing high infrastructure investment and prohibitive expenditure on consumables. The commercial kits used for nucleic acid extraction and DNA/RNA library preparation alone easily cost over 200 USD per sample, not to mention the sequencing cost. This is particularly problematic in resource-poor areas. Furthermore, the complex procedures used in sample preprocessing, host depletion and/or pathogen enrichment make these methods difficult to replicate in most clinical laboratories.

With these limitations in mind, we aimed to develop an easy-to-perform mNGS assay with minimal reliance on commercial kits and with the fewest processing steps while retaining adequate sensitivity towards most pathogen types. The resulting workflow would be affordable and widely deployable in clinical settings. In our protocol, total nucleic acids were semi-automatically extracted by chaotropic solutions and purified by magnetic beads using in-house solutions and primary reagents ordered in bulk. In addition, we also developed an efficient library preparation protocol for nanogram levels of RNA based on the template-switching properties of some reverse transcriptases^16^. This in-house method dramatically reduced the cost of RNA sequencing (~ 100 USD/sample for Illumina sequencing, ~ 300 USD for Nanopore sequencing). Moreover, we confirmed its sensitivity towards RNA viruses (< 6.4 × 10^2^ EID50 for influenza A virus) to be at least comparable to that of reported methodologies^31^. It was also found to be sensitive enough for detecting a series of RNA viruses, including human rhinovirus, human coronavirus, and HIV-1. Our assay performed well in identifying DNA/RNA viruses in our validation test set. Indeed, we quickly utilized our methodology in response to the COVID-19 outbreak. The sequencing results showed 96.4% sensitivity on qRT-PCR-confirmed COVID-19 clinical samples, and 35.7% of them yielded > 90% genome coverage (unpublished data).

Compared with the widely used sequencing-by-synthesis platforms (Illumina, Ion Torrent and PacBio sequencing), the more recently commercialized electric current sensing method (e.g., Oxford Nanopore) offers significant advantages in terms of speed and read length, making real-time data analysis feasible^32,33^. Hence, it is suitable in the context of genomic sequencing of microbes that are important to public health, as well as in the diagnosis of infectious diseases^34^. This approach has been increasingly used for molecular epidemiological research on emerging infectious diseases^35–37^. In recognition of its potential, we developed a Nanopore-compatible RNA library preparation protocol based on the SMARTer-seq principle. We tested the applicability of the workflow on RNA and DNA viruses and realized same-day reporting (< 8 h) from sample to sequencing data.

Our study still has a number of limitations. Additional workflow improvements are still needed and are underway in several aspects. First, targeted amplification of the 16S rRNA gene could provide the accuracy and sensitivity required for the identification of clinically important bacteria across species and genera^38,39^. It dramatically reduces the need to eliminate human reads and increases sensitivity, especially for samples with high host cellular content. The development of Nanopore protocols for targeted sequencing of bacterial and fungal rRNA sequences would be complementary to our current method. Second, validation of Nanopore sequencing in clinical samples and evaluation of its detection limit compared to that of Illumina sequencing is necessary. Third, better sensitivity and genome coverage could be achieved by incorporating a targeted sequence capture panel^40^, although retaining assay simplicity would be a challenge. Finally, improved sequence analytics that are efficient, bias free and rigorously validated would ensure reproducibility of reports.

In summary, a simplistic, low-cost NGS workflow that realized time- and labour-saving conversion from clinical samples to Illumina and Nanopore libraries was developed. This protocol could significantly lower the technical and economic barriers for clinical laboratories to deploy such techniques, especially in resource-poor regions.

## Methods

### Ethics statement

This study was approved by the Shanghai Public Health Clinical Center Ethics Committee. All experimental protocols involving humans were in accordance with the guidelines of the Declaration of Helsinki. Informed consent was obtained from all enrolled patients.

### Sample collection and study subjects

HBV-positive serum, human adenovirus type 7 (AdV7) and human adenovirus type 3 (AdV3) were isolated and collected in our previous studies^41,42^. Influenza A virus (A/Puerto Rico/8/1934 H1N1) (PR8 for short) was provided by Prof. Zejun Li (Shanghai Veterinary Research Institute). Eleven clinical throat swab or sputum samples that had been tested for 41 respiratory pathogens (human adenoviruses, human bocavirus, human herpesviruses, influenza A, influenza B, human parainfluenza viruses, coronaviruses, rhinovirus, enteroviruses, Haemophilus influenzae, etc.) using 384-well pre-configured TaqMan real-time PCR array cards (#4,398,986, Thermo Fisher) were used to validate the clinical performance of our mNGS method. Another 20 CSF samples taken from patients with AIDS-related meningitis or encephalitis with suspected infections were used to test our method.

### Nucleic acid extraction

Total nucleic acids (TNAs) were extracted by magnetic beads according to previously published papers with some modifications^43^. Two hundred microlitres of 1.5 × guanidinium isothiocyanate (GITC) lysis buffer (6 M GITC, 75 mM Tris–HCl (pH 7.6–8.0), 3% sarkosyl, 30 mM EDTA) was added to a 100-μL sample. Glass beads were added into the tubes. The samples were then sealed and subjected to bead beating on a Bioprep-24R homogenizer (Allsheng, China) at 4 °C and 4000 rpm for 30 s 4 times with an interval of 30 s. After homogenization, the samples were briefly centrifuged (13,000 × g, 3 min, 4 °C) and used for automatic TNA extraction on an Auto-Pure20B Nucleic Acid Purification System (Allsheng, China). The system can perform 20 sample extractions in the same run, which takes approximately 40 min. Briefly, the extraction process was as follows: 300 μL of homogenized samples was transferred into the sample well of the extraction tray for automatic TNA extraction. Four hundred microlitres of isopropanol and 4 μL of carboxyl-coated magnetic beads (16,960,972, GE, USA) diluted in 200 μL of TE buffer (10 mM Tris HCl, 1 mM EDTA, pH8.0) were added to the sample. The samples were gently mixed for 7 min. Then, the beads were washed, in order, with 500 μL of isopropanol, 800 μL of 80% ethanol and 800 μL of 80% ethanol. After the magnetic beads were dried for 7 min in air, the extracted TNA was dissolved in 50 μL of pure water. The whole TNA extraction process took approximately 1 h, with approximately 20 min of hands-on time. The extracted total nucleic acids were collected and split into aliquots for subsequent DNA and RNA library preparation for Illumina or Nanopore sequencing.

### Illumina library preparation and sequencing

DNA and RNA libraries were constructed independently for each clinical sample. For DNA libraries, we used a Tn5 transposase-based tagmentation method (TruePrep DNA Library Prep Kit V2 for Illumina, TD503-02, Vazyme Biotech Co., Ltd.) followed by PCR (13–16 cycles) with indexed primers (TruePrep Index Kit V2, Vazyme Biotech Co., Ltd.). For RNA libraries, we initially used the commercial SMARTer Universal Low Input RNA Kit (TaKaRa) to test its efficiency in cDNA library construction based on the template switching mechanism. We then developed our own SMARTer-seq protocol by modifying the SMART-seq2 protocol^17^. Briefly, **4** μL of TNA was mixed with 0.5 μL of SMARTer RT primer (10 μM, 5′- ACACTCTTTCCCTACACGACGCNNNNNN-3′), 2 μL of 5 × Maxima H Minus RT Buffer, 1 μL of MgSO4 (100 mM) and 0.25 µL of Recombinant RNase Inhibitor (40 U/μL, TaKaRa), denatured at 65 °C for 5 min and then immediately placed on ice. Then, 1 μL of dNTP mix (10 mM), 0.5 µL of TSO (20 μM; ACACTCTTTCCCTACACGACGCrGrG + G, where rG represents ribonucleotide, and + G represents locked nucleic acid), 0.5 µL of Maxima H Minus Reverse Transcriptase (200 U/μL, Thermo Fisher) and 0.25 µL of RNase Inhibitor were added. Reverse transcription was carried out by incubating at 25 °C for 10 min and 50 °C for 30 min, followed by inactivation by incubation at 85 °C for 5 min. The volume after first-strand cDNA synthesis was 10 μL. Then, 8 μL of first-strand cDNA was used for PCR amplification. Twenty microlitres of 2 × Phanta Max Master Mix (Vazyme Biotech Co., Ltd.), 0.2 μL of SINGV PCR primer (10 μM, 5′- ACACTCTTTCCCTACACGACGC -3′) and 11.8 μL of nuclease-free water were added to a final reaction volume of 40 μL. The reaction was incubated at 95 °C for 3 min and then cycled 25 times as follows: 95 °C for 20 s, 67 °C for 15 s, and 72 °C for 2 min. PCR products were purified using a 1:1 ratio (v/v) of VAHTS DNA Clean Beads (Vazyme Biotech C., Ltd), with the final elution performed in 20 μL of nuclease-free water. The extracted DNA products were quantified using the ds DNA HS Assay Kit (Thermo Fisher) on a Qubit 3.0 Fluorometer (Thermo Fisher). Approximately 5 ng of amplified product was used for library construction using the TruePrep DNA Library Prep Kit V2 for Illumina (TD503-02, Vazyme Biotech Co., Ltd.). The amplified product (13–15 cycles) was purified using AMPure XP beads. Sequencing was performed on a NovaSeq 6000 with a 2 × 150-bp paired-end sequencing protocol, and 10 to 150 million reads were generated for each sample. For each batch of samples, a pure water control or optionally a negative sample control (specific pathogen free) was included and analysed in parallel.

### Nanopore library preparation and sequencing

An influenza A strain, PR8, and two adenovirus B (AdV3 and AdV7) stocks were analysed by Nanopore sequencing as representative RNA and DNA viruses. TNA were extracted from these samples.

For influenza A H1N1, viral RNA was reverse transcribed, and SINGV PCR was amplified (35 cycles) by the SMARTer-Seq protocol. The amplified products were purified using 0.6 × volume of VAHTS DNA Clean Beads (Vazyme Biotech Co., Ltd.). The extracted DNA products were quantified using the ds DNA HS Assay Kit on a Qubit 3.0 Fluorometer. Approximately 1 μg of amplified product was used for library construction using the SQK-LSK108 kit (Oxford Nanopore Technologies). Library construction was performed according to the manufacturer’s instructions. For adenovirus B, TNA extracted from AdV3 and AdV7 stocks was used for library construction using the SQK-RPB004 kit (Oxford Nanopore Technologies) with 25 cycles of amplification. Each sample was amplified with a unique barcode primer provided in the kit.

Libraries were sequenced on the MinION platform using R9 flow cells. The H1N1 sample was first loaded onto the R9 flow cell. After sequencing the H1N1 virus for 24 h, we washed the sequencing flow cell using the Wash Kit EXP-WSHSP2 (Oxford Nanopore Technologies) following the manufacturer’s protocol and reloaded it with barcoded libraries generated with AdV 3 and 7 DNA. MinION was run for up to 24 h for each group of samples, and the first 2 h of data were used for data processing and alignment to evaluate the possibility of quick pathogen identification.

### Data analysis

Data analysis was performed in a Ubuntu20.04.1 LTS 64 bit system based on a workstation equipped with an Intel Xeon W-2133 CPU 3.6 GHz × 12, with 256 GB of memory and a 3.0-TB hard drive. The data were transferred to external hard drives for long-term storage.

Paired-end 150-base-pair sequences generated by Illumina sequencing were processed for classification and mapping using our rapid computational pathogen detection pipeline (Fig. 1b). First, reads were preprocessed by Fastp v 0.20.0^44^ for trimming of adapters and removal of low-quality (q < 20), short (less than 30) and low-complexity sequences. Second, the qualified reads were mapped to the human reference genome using bowtie2 v 2.3.5^45^ and samtools v 1.9^46^ to remove human sequences. Third, the remaining unique, nonhuman sequences were taxonomically classified against the viral genomes or NCBI nucleotide sequences (NT database, 98 GB) using Centrifuge v 1.0.4^47^. Fourth, the unique, nonhuman reads were mapped against the curated RVDB viral sequence database^48^ or the reference sequence of the specific pathogen selected from the Centrifuge output summary using bowtie2 (v2.3.5). Genome alignments and genome coverage (%) were visualized using Tablet (v19.09.03)^49^. The sequencing data were analysed in terms of the numbers of filtered reads, the number of reads aligned to the species-specific sequence, the number of mapped reads per million filtered reads, genome coverage (%) and coverage depth (average and maximum). For Illumina sequencing data, the analysis took approximately 2 h 20 samples.

For Nanopore sequencing data, raw FAST5 files from the MinION instrument were base-called by Guppy (v 3.2.4). Base-called FASTQ files were processed by filtlong software (v0.2.0) for removal of low-quality (q > 7) and short (less than 100) sequences. The qualified reads were then aligned to the curated RVDB viral sequence database using minimap2 (v 2.17-r941). Mapped reads were exported to a bam file using samtools and visualized using Tablet. Identification of pathogens by minimap2-based pathogen-specific sequencing alignment could be performed within 10 min after real-time sequencing.

All the Illumina and Nanopore sequencing raw data were deposited in the Sequence Read Archive (SRA) database with accession codes: PRJNA692001 (https://www.ncbi.nlm.nih.gov/sra/PRJNA692001).

### Positive reporting threshold and assay controls

For each batch of Illumina sequencing libraries, the “no template” control (NTC), i.e., nuclease-free water, was processed in parallel with samples, and the resulting reads were used as background references. Pathogen reporting threshold criteria were established to minimize false-positive results from contaminating microbial sequences. Identified RNA viruses were reported based on analysis of RNA mNGS libraries, whereas DNA viruses, bacteria, fungi, and parasites were reported based on analysis of a DNA or RNA library, depending on the abundance of the pathogen-mapped reads. For viruses, the threshold criteria were based on the detection of non-overlapping reads from ≥ 3 distinct genomic regions. For the identification of bacteria, fungi, and parasites, a reads per million (RPM) ratio metric (RPM-r) was used, defined as RPM-r = RPM_sample_/RPM_NTC_, with the minimum RPM_NTC_ set to 1^27^. A minimum threshold of RPM-r ≥ 10 was designated for reporting the detection of a bacterium, fungus, or parasite.
